# Antioxidant Treatment in Male Mice Prevents Mitochondrial and Synaptic Changes in an NMDA Receptor Dysfunction Model of Schizophrenia

**DOI:** 10.1523/ENEURO.0081-17.2017

**Published:** 2017-08-17

**Authors:** Aarron Phensy, Christopher Driskill, Karen Lindquist, Lan Guo, Vivek Jeevakumar, Bryan Fowler, Heng Du, Sven Kroener

**Affiliations:** 1 School of Behavioral and Brain Sciences, The University of Texas at Dallas, Richardson, Texas 75080; 2 Department of Biological Sciences, The University of Texas at Dallas, Richardson, Texas 75080

**Keywords:** GABA, glutamate, prefrontal cortex, glutathione, oxidative stress, mitochondria

## Abstract

Glutamate theories of schizophrenia suggest that the disease is associated with a loss of NMDA receptors, specifically on GABAergic parvalbumin-expressing interneurons (PVIs), leading to changes in the excitation–inhibition balance in the prefrontal cortex (PFC). Oxidative stress contributes to the loss of PVI and the development of schizophrenia. Here, we investigated whether the glutathione precursor *N*-acetyl cysteine (NAC) can prevent changes in synaptic transmission at pyramidal cells and PVIs that result from developmental NMDAR blockade and how these changes are related to mitochondrial dysfunction in the PFCs of mice. Perinatal treatment with ketamine induced persistent changes in the reduced glutathione/oxidized glutathione (glutathione disulfide) ratio in the medial PFC, indicating long-lasting increases in oxidative stress. Perinatal ketamine treatment also reduced parvalbumin expression, and it induced a decline in mitochondrial membrane potential, as well as elevations in mitochondrial superoxide levels. At the level of synaptic function ketamine reduced inhibition onto layer 2/3 pyramidal cells and increased excitatory drive onto PVI, indicating long-lasting disruptions in the excitation–inhibition balance. These changes were accompanied by layer-specific alterations in NMDAR function in PVIs. All of these changes were mitigated by coadministration of NAC. In addition, NAC given only during late adolescence was also able to restore normal mitochondria function and inhibition at pyramidal cells. These results show that ketamine-induced alterations in PFC physiology correlate with cell type-specific changes in mitochondria function. The ability of NAC to prevent or restore these changes supports the usefulness of antioxidant supplementation in the treatment of schizophrenia.

## Significance Statement

Previous reports have shown that oxidative stress reduces parvalbumin expression in the PFC, which correlates with schizophrenia-like behavioral deficits. Both the loss of parvalbumin and the behavioral deficits can be prevented by modulation of glutathione levels, including *N*-acetyl cysteine (NAC) supplementation. Here we examine for the first time how NAC affects ketamine-induced changes in synaptic transmission in the PFC network. Our report is also the first to demonstrate cell type-specific changes in mitochondrial ROS production that result from developmental ketamine treatment and how these can be affected by NAC supplementation at various stages during development.

## Introduction

Schizophrenia is a neurodevelopmental disorder in which genetic risk factors and early life stressors converge. Blockade of NMDA receptors (NMDARs) induces behavioral changes in animals (Chatterjee et al., 2012; Jeevakumar et al., 2015; Phensy et al., 2017) that mimic the positive and negative symptoms, as well as cognitive deficits observed in schizophrenic patients (Lahti et al., 1995; Malhotra et al., 1997; Krystal et al., 2002). Such treatment also reduces the levels of the GABA-synthesizing enzyme GAD67 and the number of parvalbumin-expressing interneurons (PVIs; Braun et al., 2007; Coleman et al., 2009; Jeevakumar and Kroener, 2016). These changes replicate findings from postmortem studies in schizophrenic patients suggesting that dysfunctions of GABAergic interneurons are a core feature of schizophrenia (Lewis et al., 2005). While the processes that lead to these changes are still poorly understood, accumulating evidence suggests that oxidative stress is an important factor in the pathophysiology of the disease. Both peripheral tissue and neurons in the CNS of schizophrenic patients show oxidative stress and abnormal levels of antioxidants, including glutathione, which provide endogenous protection against reactive oxygen species (ROS; Yao and Keshavan, 2011). Mitochondria are the major source of ATP and ROS production and play a critical role in regulating intracellular redox balance. The close association of mitochondrial dysfunction in energy production and ROS regulation with negative symptoms and cognitive deficits in schizophrenia suggests that mitochondrial defects play an important role in the pathogenesis of the disease (Ben-Shachar and Laifenfeld, 2004; Rajasekaran et al., 2015). PVIs possess a large number of mitochondria and a unique metabolic profile that renders them particularly susceptible to external stressors during development (Kann and Kovács, 2007). NMDAR dysfunction in PVI causes the downregulation of GABA release and subsequent disinhibition of pyramidal cells (Zhang et al., 2008; Kjaerby et al., 2014; Jeevakumar and Kroener, 2016), which contributes to excessive glutamate release (Sorce et al., 2010) and to further oxidative stress (Behrens et al., 2007) and glutathione deficits (Papadia et al., 2008; Radonji et al., 2010). For these reasons, antioxidants hold considerable potential for the treatment or prevention of pathologies in schizophrenia (Do et al., 2015). Supplementation with the glutathione precursor *N*-acetyl cysteine (NAC) can prevent the changes in PV expression and a range of cognitive and behavioral deficits that occur in animal models of schizophrenia (Cabungcal et al., 2014, 2013; Phensy et al., 2017). However, while a role for oxidative stress in PVI dysfunction is well established (Behrens et al., 2007; Cabungcal et al., 2013, 2014), specific changes in mitochondria that may contribute to these effects have received little attention. Moreover, how NAC affects the synaptic physiology of PVI is still unknown. Here, we tested whether antioxidant treatment with NAC can prevent deficits in prefrontal cortex (PFC) function induced by perinatal NMDAR blockade with ketamine (KET) and how restoring redox regulation affects mitochondrial function in PVI. Developmental ketamine treatment altered the reduced glutathione (GSH)/oxidized glutathione (GSSG) ratio, reduced PV immunoreactivity, and affected mitochondrial membrane potential and ROS production in adult mice, leading to aberrant synaptic transmission between pyramidal cells and PVIs in the medial PFC (mPFC). Mitochondrial dysfunction was more pronounced in PVIs than in pyramidal cells, and the changes in glutamatergic inputs to PVIs showed layer-specific differences. All of these changes were prevented by NAC. These results show that developmental ketamine treatment induces long-lasting changes in the physiology of the PFC. These changes correlate with mitochondrial deficits that are exacerbated in PVIs. Antioxidant treatment with NAC prevents these changes, thus preserving normal prefrontal cortical function.

## Materials and Methods

### Animals and *in vivo* drug treatment

Male mice of the G42 line (CB6-Tg[Gad1-EGFP]G42Zjh/J; The Jackson Laboratory; RRID:IMSR_JAX:007677), which express GFP in PVIs, were used for the experiments. Littermates were assigned to one of the following four groups: saline-control (SAL-SAL); ketamine followed by saline (KET-SAL); ketamine followed by NAC (KET-NAC); or saline followed by NAC (SAL-NAC). Starting on postnatal day 5 (P5), all animals received subcutaneous injections of either saline or NAC (0.1% in saline, 10 µl/g body weight; Sigma-Aldrich) until they were weaned at P21. In addition, on P7, P9, and P11 all animals received subcutaneous injections of either saline or a subanesthetic dose of ketamine (30 mg/kg; Ketathesia HCl, Henry Schein) to induce NMDAR dysfunction. After weaning, animals in the NAC groups continued to receive NAC (0.1%) in their drinking water until they were killed. Experiments were performed using adult animals (older than P90). For experiments that examined the effects of NAC treatment during late adolescence, animals received injections of either ketamine or saline on P7, P9, and P11 and then received NAC (0.1%) in their drinking water starting on P35 for 3 weeks. Experiments were performed starting after P60. All procedures were approved by the Institutional Animal Care and Use Committee of The University of Texas at Dallas.

### Immunohistochemistry

Animals were perfused with saline followed by 4% paraformaldehyde (PFA; Fisher Scientific) in 0.12 m PBS, at 4°C and pH 7.4. Brains were postfixed in PFA with 30% sucrose for 1 h and then transferred to 30% sucrose (Sigma-Aldrich) in PBS for 18 h at 4°C. Coronal slices (40 μm) were cut on a freezing microtome. Free-floating sections were incubated in rabbit anti-parvalbumin (1:2000 working dilution; Swant; RRID:AB_10000344) in PBS and 0.3% Triton X (Sigma-Aldrich) for 36 h at 4°C. Sections were washed three times for 10 min each in PBS before they were incubated in secondary DyLight 594 Goat Anti-rabbit (1:1000 working dilution; Jackson ImmunoResearch) in PBS and 0.3% Triton X. Sections were washed, mounted, and coverslipped using Prolong Gold Antifade with DAPI (Thermo Fisher Scientific). For each animal, a minimum of four sections including the prelimbic and infralimbic regions of the PFC were imaged on a confocal microscope (FluoView 1000, Olympus) at 20× magnification. The number of PV^+^ cells were hand counted in ImageJ (National Institutes of Health), and DAPI-labeled cells were counted using the thresholding function in ImageJ to obtain the percentage of total PV^+^ cells among all DAPI-labeled cells. PV expression in the treatment groups was normalized to the saline-treated control group to calculate the percentage change in PV.

### Analysis of glutathione levels

GSSG, GSH, and total glutathione were measured following the manufacturer instructions (glutathione detection kit, catalog #ADI-900-160, Enzo Life Sciences). In brief, animals were killed and the medial PFC (containing the infralimbic and prelimbic cortex) was dissected and homogenized in ice-cold 5% (w/v) meta-phosphoric acid (20 ml/g tissue), followed by centrifugation at 12,000 × *g* for 10 min at 4ᵒC. The resultant supernatant was collected for glutathione detection. For the measurement of GSSG and total glutathione, 2 m 4-vinylpyridine was added to the samples at a dilution of 1:50 (v/v). The samples were then incubated for 1 h at room temperature to derivatize reduced glutathione. Afterward, the samples were diluted in the reaction mix buffer. The reaction was observed by immediately and continuously recording changes at an optical density of 405 nm by using a microplate reader (Biotek) for a total of 15 min at 1 min intervals. The concentrations of total, oxidized, and reduced glutathione were normalized to the original wet weight of the tissue.

### *In situ* detection of mitochondrial membrane potential and mitochondrial superoxide levels

To analyze changes in mitochondrial membrane potential, acute brain slices (150 µm) of the prelimbic and infralimbic regions of the PFC were prepared from animals in all treatment groups at P90 to P120 and perfused with oxygenized recording artificial CSF (ACSF) containing tetramethylrhodamine methyl ester (TMRM; 200 nm; Thermo Fisher Scientific) for 30 min, followed by a brief wash in oxygenized recording ACSF. Unfixed slices were immediately mounted and coverslipped, and images were collected on an inverted confocal microscope (Eclipse Ti-E, Nikon). TMRM intensity in GFP^+^ PVIs located 10–15 µm below the slice surface was analyzed using Nikon NIS Advanced Research software. Similarly, to analyze mitochondrial superoxide levels, acute brain slices (150 µm) were prepared at P90 to P120 and perfused for 30 min with oxygenized recording ACSF containing MitoSox Red (2 µm; Thermo Fisher Scientific), a fluorescent indicator of mitochondrial superoxide. Brain slices were washed and fixed in 4% PFA at 4ᵒC overnight. To identify PVIs and pyramidal neurons, respectively, the slices were double labeled with rabbit anti-PV (1:2000 working dilution; Swant; RRID:AB_10000344) and mouse anti-CamKII-α (1:300 working dilution; Cell Signaling Technology) in PBS and 0.3% Triton X. Sections were washed three times for 10 min each in PBS before they were incubated with Alexa Fluor 488-conjugated goat anti-rabbit IgG (1:500 working dilution; catalog #11029, Life Technologies) and Alexa Fluor 647-conjugated goat anti-mouse IgG (1:1000 working dilution; catalog #4410S, Cell Signaling Technology) for 1 h at room temperature. Images of MitoSox Red, PV, and CamKII-α staining were obtained on a Nikon confocal microscope. For a cell type-specific analysis of mitochondrial superoxide production *z*-stacked confocal images were obtained and 3D reconstructions of cells located 10–15 µm from the slice surface were used to colocalize MitoSox Red with PV and CamKII-α staining (NIS Advanced Research, Nikon).

### Electrophysiology

Electrophysiological experiments used GFP^+^ hemizygous mice. Mice were overdosed with urethane (3 g/kg body weight; Fisher Scientific) and decapitated. Brains were extracted, and coronal sections (350 µm) of the frontal cortex were cut on a vibratome (VT1000S, Leica) in ice-cold oxygenated (95% O_2_, 5% CO_2_) ACSF containing the following (in mm): 110 choline (Sigma-Aldrich), 25 NaHCO_3_ (Fisher Scientific), 1.25 NaH_2_PO_4_ (Fisher Scientific), 2.5 KCl (Sigma-Aldrich), 7 MgCl_2_ (Sigma-Aldrich), 0.5 CaCl_2_ (Sigma-Aldrich), 10 dextrose (Fisher Scientific), 1.3 l-ascorbic acid (Fisher Scientific), and 2.4 Na^+^-pyruvate (Sigma-Aldrich), bubbled with 95% O_2_/5% CO_2_. Slices were incubated for 1 h at 35ᵒC in ACSF containing the following (in mm): 126 NaCl (Fisher Scientific), 25 NaHCO_3_, 1.25 NaH_2_PO_4_, 2.5 KCl, 2 MgCl_2_, 2 CaCl_2_, 10 dextrose, 2.4 Na^+^-pyruvate, and 1.3 l-ascorbic acid, bubbled with 95% O_2_/5% CO_2_. Whole-cell voltage-clamp recordings were obtained from pyramidal cells and PVIs in the prelimbic and infralimbic cortex at room temperature using oxygenated recording ACSF containing the following (in mM): 120 NaCl, 2.5 KCl, 1.25 NaH_2_PO_4_, 25 Na_2_HCO_3_, 10 dextrose, 2 CaCl_2_, and 2 MgCl_2_, 2.4 Na^+^-pyruvate, and 1.3 l-ascorbic acid. For voltage-clamp recordings, electrodes (WPI; 3–5 MΩ open tip resistance for pyramidal cells, 6–8 MΩ for interneurons) were filled with the following (in mM): 130 CsCl (Sigma-Aldrich), 20 tetraethylammonium chloride (Sigma-Aldrich), 10 HEPES (Sigma-Aldrich), 2 MgCl_2_, 0.5 EGTA (Sigma-Aldrich), 4 Mg^2+^-ATP (Sigma-Aldrich), 0.3 Lithium-GTP (Sigma-Aldrich), 14 phosphocreatine (Sigma-Aldrich), and 2 QX-314 bromide (Tocris Bioscience). Recordings were performed on an Axon Multiclamp 700B amplifier (Molecular Devices), and data were acquired and analyzed using AxoGraph X (AxoGraph Scientific). Access resistance was monitored throughout the recording, and a <20% change was deemed acceptable. Theta-glass pipettes (Warner Instruments) connected to a stimulus isolator (WPI) were used for focal stimulation of synaptic potentials. Glutamatergic events were isolated by bath application of the chloride channel blocker picrotoxin (75 µm; Sigma-Aldrich). When recording IPSCs, AMPA receptor-mediated events were blocked using 20 µm CNQX (6-cyano-7-nitroquinoxaline-2,3-dione; Sigma-Aldrich). Miniature events were recorded in the presence of 1 µm tetrodotoxin (Alomone Labs) in the bath. The frequency and amplitude of spontaneous and miniature postsynaptic currents were measured from 200 s of continuous recording using MiniAnalysis (Synaptosoft) with a threshold set at two times the rms baseline noise. To study ketamine-induced changes in glutamatergic currents, we calculated the NMDAR/AMPAR-mediated current ratio. A compound evoked EPSC (eEPSC) was first recorded at a holding potential of +40 mV. The AMPA component of the synaptic response was then pharmacologically isolated by bath application of 10 µm (±)-3-(2-carboxypiperazin-4-yl)propyl-1-phosphonic acid (CPP; Sigma-Aldrich). A minimum of 15 traces of the compound eEPSC and the AMPA component, respectively, were collected and averaged. The averaged AMPA current was digitally subtracted from the compound response to yield the NMDAR current. The peaks of the isolated AMPA and NMDA responses were divided to calculate the NMDAR/AMPAR ratio.

### Data analysis

Differences among the treatment groups in all experiments were assessed with one-way or two-way ANOVAs using SPSS (version 22, IBM). *Post hoc* comparisons used Bonferroni tests to determine statistical significance between groups. For immunohistochemical experiments, *n* indicates the number of animals per condition, while in electrophysiological experiments *n* indicates the number of cells recorded. All data are represented as the mean ± SEM, with *p* < 0.05 being considered statistically significant.

## Results

### *N*-acetyl cysteine prevents ketamine-induced changes in the ratio of reduced and oxidized glutathione

**Figure 1.**
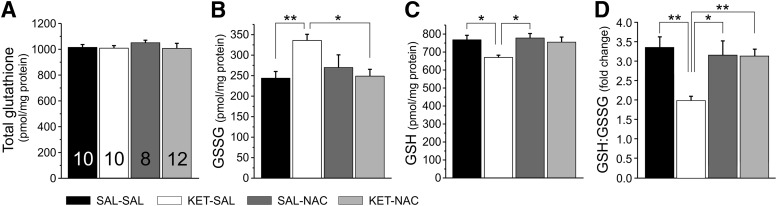
Perinatal ketamine treatment disturbed the balance between GSSG and GSH levels in the adult medial prefrontal cortex, which was prevented by NAC treatment. ***A***, Perinatal ketamine treatment did not alter the total levels of glutathione in mPFC tissue from adult mice. ***B***, ***C***, However, the levels of GSSG and GSH were shifted in opposite directions in the KET-SAL group. ***B***, ***C***, GSSG levels were significantly increased (***B***) and GSH levels were significantly decreased (***C***) in mPFC tissue following ketamine treatment. ***D***, Changes in glutathione levels expressed as the GSH/GSSG ratio. KET-SAL treated mice showed significantly reduced ratios, indicating increased oxidative stress. Concomitant NAC treatment restored the ratio to a level comparable to that of saline-treated control mice (SAL-SAL). Significance is indicated as *p ≤ 0.05 and **p ≤ 0.01, following Bonferroni correction.

GSH is one of the most important scavengers of ROS, and its ratio with GSSG can be used as a marker of oxidative stress. Acute application of ketamine increases oxidative stress, in part by reducing levels of GSH (de Oliveira et al., 2009; da Silva et al., 2010). We treated mice perinatally with ketamine and used an ELISA to detect persistent changes in the levels of GSH and GSSG in homogenates from adult mPFC and to test whether NAC can protect against potential changes in oxidative stress. Separate one-way ANOVAs showed a main effect of treatment on total levels of GSSG (*F*_(3,36)_ = 4.945, *p* = 0.006) as well as GSH (*F*_(3,36)_ = 4.016, *p* = 0.015) without altering the total levels of glutathione (*F*_(3,36)_ = 0.431, *p* = 0.732; Fig. 1*A–C*). A one-way ANOVA also showed a main effect of treatment on the GSH/GSSG ratio (*F*_(3,36)_ = 6.447, *p* = 0.001). *Post hoc* tests indicate that this effect was caused by a significant increase of the ratio in KET-SAL-treated animals, which was prevented by cotreatment with NAC throughout development (KET-NAC). Together, these data suggest persistent alterations in antioxidative defense systems in the mPFC following perinatal ketamine treatment, which can be prevented by the coadministration of NAC.

### *N*-acetyl cysteine prevents ketamine-induced loss of parvalbumin expression

**Figure 2.**
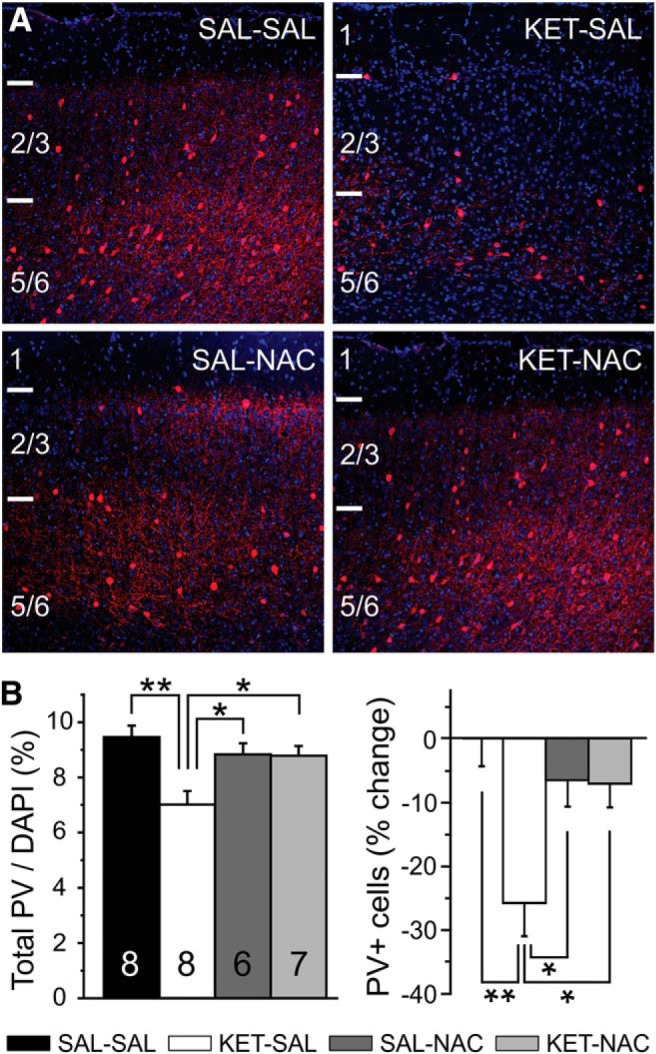
Subchronic ketamine treatment during development reduced PV immunoreactivity in adult animals, which was prevented by simultaneous treatment with NAC. ***A***, Representative confocal images of the mPFC from adult mice in the four treatment groups stained for PV (red) and DAPI (blue). ***B***, The number of PV-expressing neurons (represented as the number of PV immunoreactive cells over DAPI across layers 2/3 and layer 5) was significantly reduced in animals treated with ketamine, and this was prevented by concomitant NAC treatment. Significance is indicated as *p ≤ 0.05 and **p ≤ 0.01, following Bonferroni correction.

Reductions in PV expression are an important characteristic of several rodent models of schizophrenia (Behrens et al., 2007; Braun et al., 2007; Hikida et al., 2007; Lodge et al., 2009; Zhang et al., 2008; Steullet et al., 2017) because they mimic changes in GABAergic interneurons seen in postmortem brains of schizophrenic subjects (Lewis et al., 2005; Akbarian and Huang, 2006). A number of genetic and environmental risk factors may contribute to the abnormal development of PVIs. In addition, oxidative stress can lead to PVI impairments (for review, see Steullet et al., 2017). We used fluorescence immunohistochemistry to determine whether NAC treatment can prevent ketamine-induced reductions in PV expression in the medial PFC. Perinatal ketamine treatment led to the persistent reduction of PV expression in the adult PFC (Fig. 2). A one-way ANOVA revealed a main effect of treatment (*F*_(3,25)_ = 6.614, *p* = 0.002), with the number of PVIs (expressed as a percentage of DAPI-stained cells) significantly reduced across all cortical layers in ketamine-treated animals. Treatment with NAC significantly ameliorated this reduction in PVIs (Fig. 2*B*).

### NAC treatment protects mitochondrial function in PVIs in the PFC

**Figure 3.**
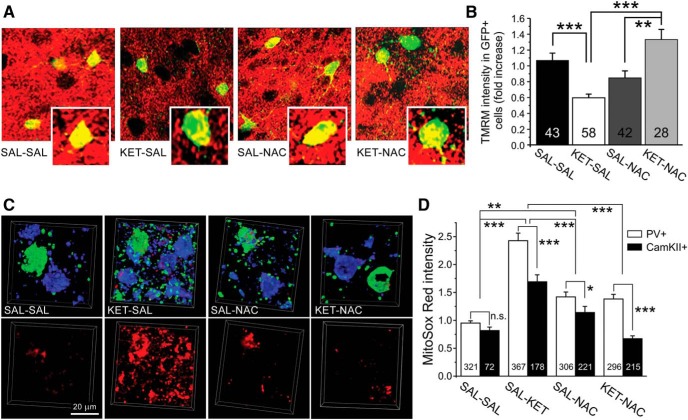
NAC protected mitochondrial function against ketamine-induced oxidative stress. ***A***, Acute brain slices of the PFC from adult mice in the four treatment groups were stained with TMRM (red) to measure ketamine-induced changes in mitochondrial membrane potential. PVIs were identified by their GFP expression (green). ***B***, PVIs from ketamine-treated animals exhibited significantly reduced TMRM intensity, which was mitigated by NAC (**p ≤ 0.01, ***p ≤ 0.001; *N* = 4-5 mice/group). ***C***, Ketamine-induced changes in mitochondrial superoxide levels as indicated by MitoSox Red staining. Acute brain slices from adult mice were stained for MitoSox Red (bottom row); after fixation, the MitoSox Red signal was colocalized with immunostaining for PV (top row, green) and CamKII-α (top row, blue), to identify PVIs and pyramidal cells, respectively. Scale bar, 20 µm. ***D***, Analysis of cell type-specific changes in mitochondrial superoxide levels in PVIs and pyramidal cells (black bars). Ketamine treatment increased mitochondrial ROS levels in both PVIs and pyramidal cells of the PFC; however, changes in PVIs were significantly larger, suggesting relatively greater vulnerability of PVIs to mitochondrial oxidative stress in response to ketamine. The application of NAC significantly ameliorated ketamine-induced mitochondrial ROS accumulation in both PVIs and pyramidal cells. NAC by itself also resulted in small elevations in ROS (*p ≤ 0.01, **p ≤ 0.01, ***p ≤ 0.001. *N* = 4–7 mice/group).

Oxidative stress is well documented in the pathophysiology of schizophrenia (Do et al., 2015). Mitochondria are the major source of ATP and ROS in neurons. Excess mitochondrial ROS production and accumulation are closely associated with mitochondrial dysfunction, resulting in oxidative stress. However, the contribution of mitochondrial deficits to oxidative stress in PVIs remains unclear. The mitochondrial membrane potential is a sensitive indicator of mitochondrial function (Perry et al., 2011). Accumulation of the fluorescent indicator TMRM is proportional to the mitochondrial membrane potential, and reduced TMRM intensity is a sensitive indicator of mitochondrial depolarization. To determine the effects of ketamine and NAC treatments on mitochondrial functional status in PVIs, we used TMRM and examined the changes in the mitochondrial membrane potential in GFP^+^ PVIs across all layers of the PFC. A one-way ANOVA showed a main effect of treatment on TMRM intensity (*F*_(3,167)_ = 13.848, *p* < 0.001). *Post hoc* analyses revealed that this was due to significantly decreased TMRM intensity in PVI from ketamine-treated mice. The ketamine-induced mitochondrial depolarization was substantially mitigated by concurrent NAC treatment (Fig. 3*B*). These results suggest that ketamine has a deleterious impact on mitochondrial oxidative phosphorylation (OXPHOS) in PVIs, which can be prevented by NAC.

Because there is a strong correlation between mitochondrial OXPHOS deficiency and ROS production, we next examined mitochondrial ROS levels directly by staining with MitoSox Red, a fluorescent indicator of mitochondrial superoxide abundance. Furthermore, to investigate whether PVIs and pyramidal cells show different vulnerabilities to ketamine-induced mitochondrial oxidative stress, we compared the relative changes in mitochondrial ROS levels in PVIs and pyramidal cells. Therefore, we first stained for MitoSox Red in acute brain slices and after fixation performed immunohistochemistry for PV and CamKII-α to label PVIs and pyramidal cells, respectively (Fig. 3*C*,*D*). For the cell type-specific quantitative analysis, the measures of mitochondrial ROS in CaMKII-α^+^ and PV^+^ cells from all four treatment groups were normalized to the MitoSox Red intensity in vehicle-treated (SAL-SAL) PVIs. A two-way ANOVA revealed a main effect of treatment (*F*_(3,1968)_ = 46.461, *p* < 0.001) and an interaction of treatment × cell type (*F*_(3,1968)_ = 3.180, *p* = 0.023). *Post hoc* analyses showed that both PV^+^ and CaMKII^+^ cells from ketamine-treated animals showed increased MitoSox Red intensity when compared with cells in the other treatment groups, suggesting that ketamine causes excess generation and accumulation of mitochondrial ROS (Fig. 3*D*). Pairwise comparisons also revealed that ketamine treatment induced significantly larger relative increases in mitochondrial superoxide levels in PVIs than in pyramidal cells, suggesting a relative increased vulnerability of PVIs to the effects of developmental NMDAR blockade. It is noteworthy that in the vehicle-treated group the MitoSox Red intensity of the two cell types was comparable (Fig. 3*D*). The ketamine-induced mitochondrial ROS elevation was markedly suppressed by the administration of NAC. However, NAC by itself (SAL-NAC) also increased mitochondrial ROS in PVIs (Fig. 3*D*). Together, these results show that ketamine disrupts mitochondrial function and promotes mitochondrial ROS production in both PVIs and pyramidal cells, but the relative magnitude of this change is larger in PVIs. Importantly, all ketamine-associated mitochondrial defects were greatly attenuated by the application of NAC.

### NAC treatment prevents disinhibition of layer 2/3 pyramidal cells

**Figure 4.**
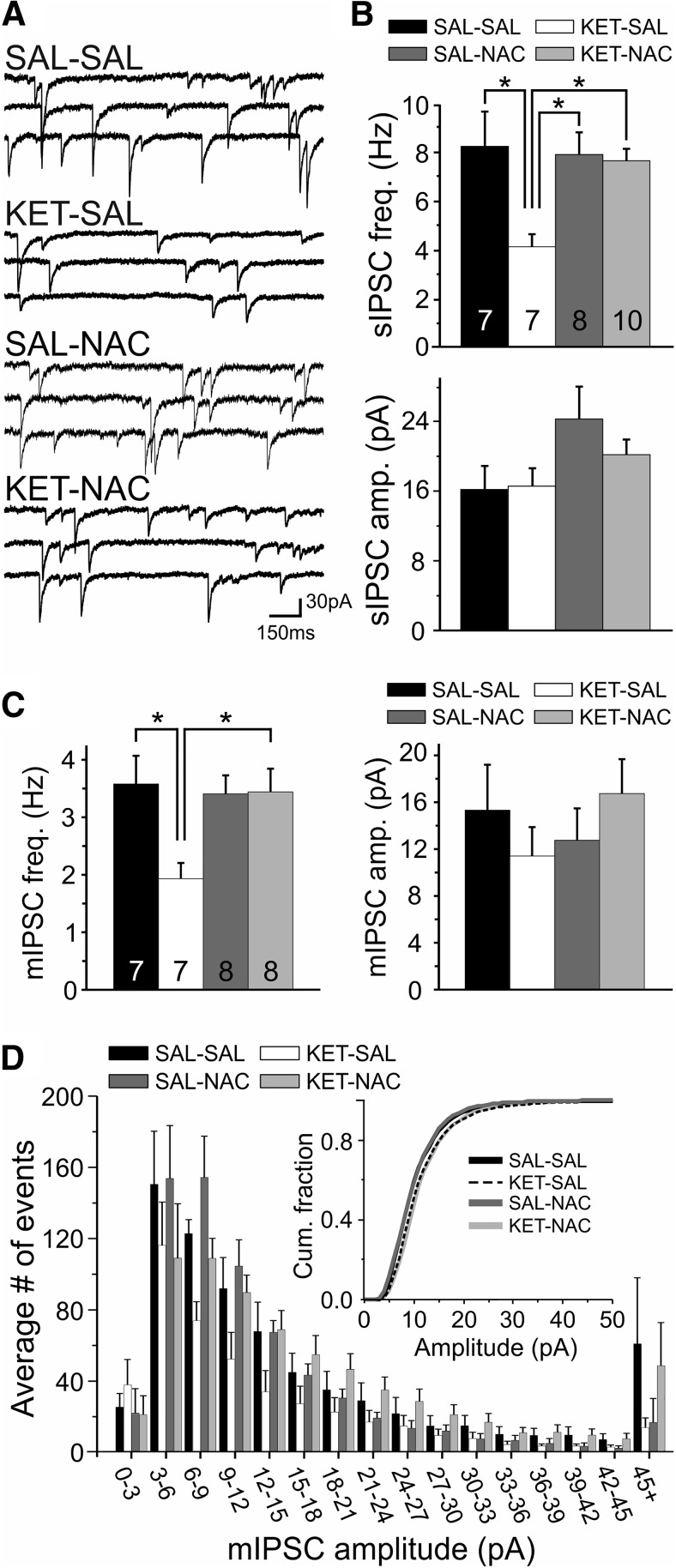
Developmental ketamine treatment leads to disinhibition of layer 2/3 pyramidal neurons in the mPFC of adult animals. *N*-acetyl cysteine treatment prevented this change. ***A***, Example traces of pharmacologically isolated sIPSCs recorded from layer 2/3 pyramidal neurons. ***B***, sIPSCs in pyramidal neurons from ketamine-treated animals (KET-SAL group) showed significant reductions in frequency (top bar graph) but no change in amplitude (bottom bar graph). Animals cotreated with NAC (KET-NAC group) did not show similar changes. ***C***, The frequency of action potential-independent mIPSCs (left bar graph) was also reduced in slices from ketamine-treated animals (KET-SAL group), suggesting that perinatal ketamine induced persistent changes in glutamate release. This change was prevented by NAC treatment (KET-NAC group). Ketamine or NAC treatment did not affect the amplitude of mIPSCs (right bar graph). ***D***, Amplitude distribution of mIPSCs from the four treatment groups. The inset shows the same data as a cumulative distribution plot, indicating that ketamine or NAC treatment did not affect mIPSC amplitude distribution. Significance is indicated as *p ≤ 0.05, following Bonferroni correction.

Impaired mitochondrial function and the loss of PV are expected to alter the synaptic function of PVIs, potentially leading to the disinhibition of cortical pyramidal cells. To assess how ketamine and NAC treatment affect the excitation–inhibition (E/I) balance in the PFC network, we first recorded spontaneous IPSCs (sIPSCs), as well as action potential-independent miniature IPSCs (mIPSCs) in layer 2/3 pyramidal cells from adult animals and compared the changes in the amplitude and frequency of events. For sIPSCs, a one-way ANOVA revealed a main effect of treatment condition on the frequency of events (*F*_(3,28)_ = 4.395, *p* = 0.012), with *post hoc* comparisons showing a selective decrease in the frequency of sIPSCs in the KET-SAL group (Fig. 4*B*). NAC prevented these ketamine-induced changes in sIPSC frequency. In contrast, the amplitude of sIPSCs was not affected by any of the treatments (*F*_(3,28)_ = 2.298, *p* = 0.099; Fig. 4*B*). To investigate whether the changes in sIPSCs result from changes in GABA release probability, we next recorded action potential-independent mIPSCs. Similar to what we observed for sIPSCs, there was a main effect of treatment on the frequency of mIPSCs (*F*_(3,26)_ = 4.183, *p* = 0.015). *Post hoc* comparisons showed that this was due to a selective reduction in mIPSC frequency in the KET-SAL group, indicating that ketamine treatment altered GABA release probability. This decrease in mIPSC frequency was also prevented by NAC treatment (Fig. 4*C*). As with sIPSCs, there were no significant differences in mIPSC amplitude among the treatment groups (*F*_(3,26)_ = 0. 743, *p* = 0.536; Fig. 4*C*). Together, these results show that NAC treatment can prevent the loss of inhibitory inputs onto layer 2/3 pyramidal neurons that following developmental ketamine treatment.

### NAC treatment prevents increased excitatory drive onto layer 2/3 PVI

**Figure 5.**
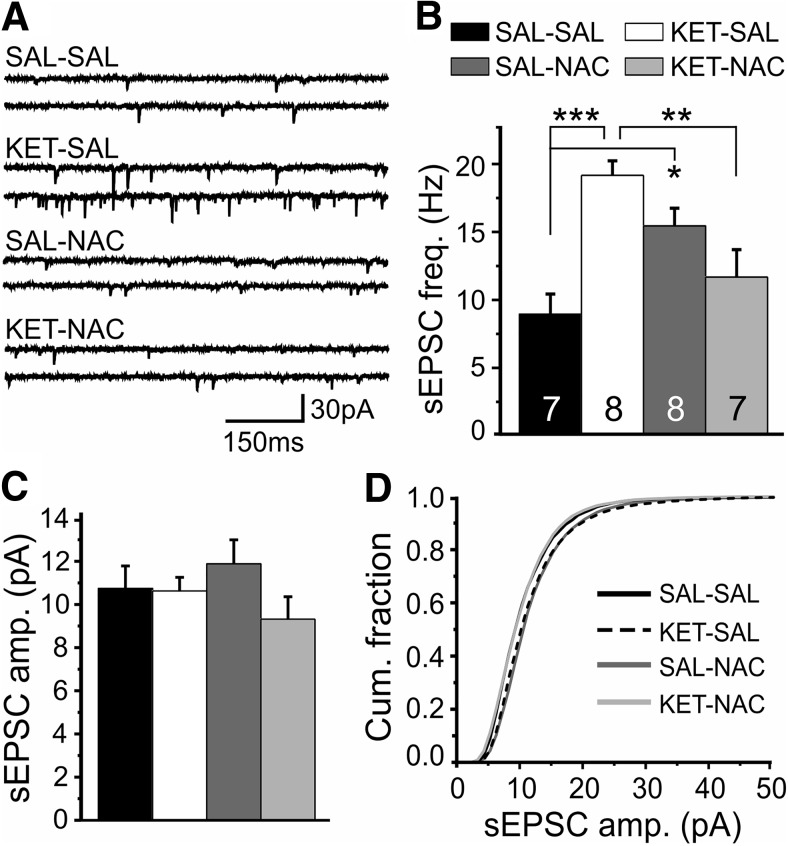
Ketamine treatment increased the frequency of excitatory inputs in PVIs in layer 2/3, which was prevented by NAC. ***A***, Representative traces of pharmacologically isolated sEPSCs recorded from layer 2/3 GFP^+^ PVIs, recorded at −65 mV. ***B***, Ketamine-treated animals showed a significant increase in the frequency of spontaneous EPSCs versus control animals. Upregulation of sEPSCs was ameliorated in animals given concomitant NAC treatment; however, NAC treatment by itself also resulted in a significant increase in sEPSC frequency. ***C***, Amplitudes of spontaneous EPSCs were not affected by any of the treatments. ***D***, A plot of the cumulative fraction of sEPSC amplitudes indicates that there was no change in the distribution of amplitudes across all groups. Significance is indicated as *p ≤ 0.05, **p ≤ 0.01, and ***p ≤ 0.001, following Bonferroni correction.

The disinhibition of pyramidal cells that results from ketamine treatment leads to increased excitatory drive at layer 5 PVIs (Jeevakumar and Kroener, 2016). While PV expression is significantly reduced in ketamine-treated animals, GFP expression in the G42 line is not similarly affected (Powell et al., 2012; Jeevakumar and Kroener, 2016). This allowed us to obtain targeted recordings of spontaneous EPSCs (sEPSCs) from GFP^+^ PVI in layers 2/3 to investigate whether NAC treatment also prevents changes in excitatory inputs onto PVIs. A one-way ANOVA showed a significant effect of treatment on sEPSC frequency in layer 2/3 PVIs (*F*_(3,26)_ = 9.144, *p* < 0.001). *Post hoc* analysis revealed that this difference resulted from a selective increase in the frequency of sEPSCs in KET-SAL animals and that NAC treatment prevented this increase (Fig. 5*B*). There was no effect of treatment on the amplitude of sEPSCs in PVIs (*F*_(3,26)_ = 1.166, *p* = 0.342; Fig. 5*C*,*D*). These data suggest that developmental ketamine treatment alters the normal E/I balance, leading to increased presynaptic excitatory activity in layer 2/3 PVIs. Restoring antioxidant balance with NAC prevents this shift.

### NAC treatment prevents ketamine-induced changes in NMDAR/AMPAR ratios at PVI

**Figure 6.**
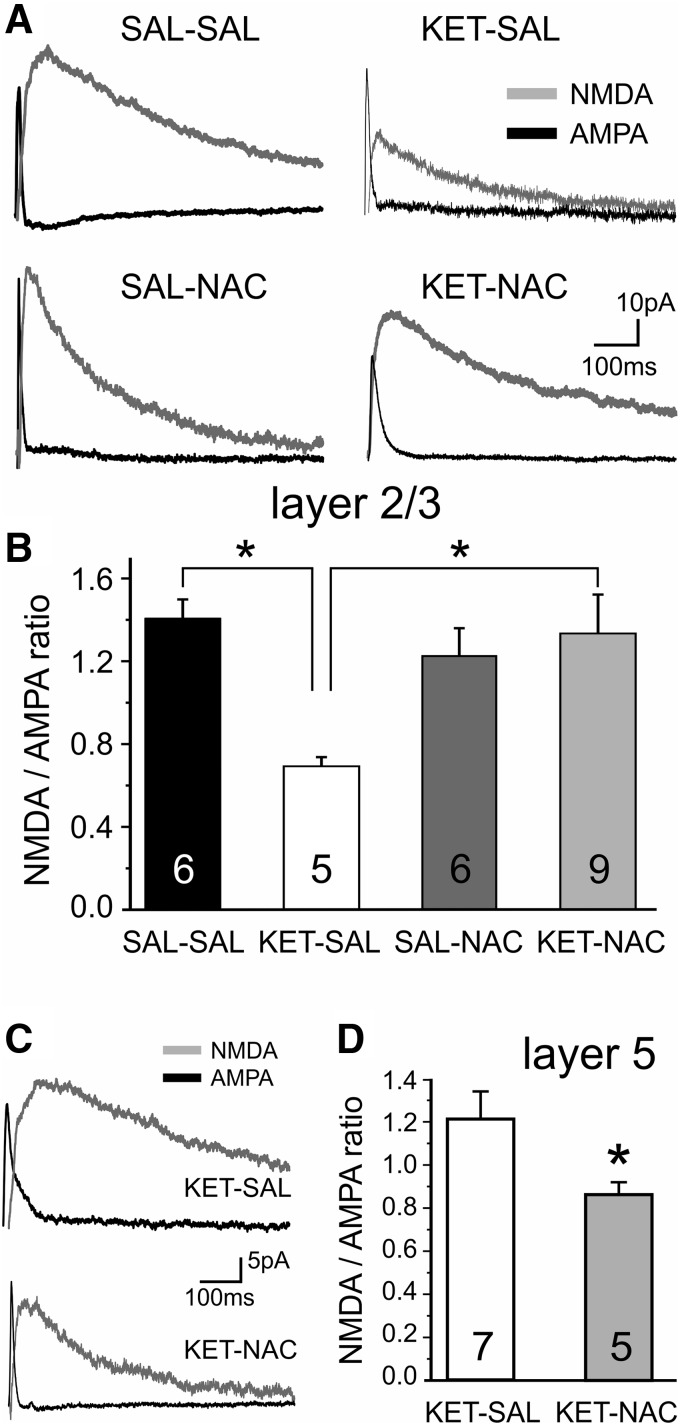
Developmental ketamine treatment resulted in laminar-specific alterations in NMDAR/AMPAR current ratios in PVIs. Perinatal ketamine treatment reduces NMDA receptor contribution at glutamatergic synapses onto PVI in superficial layers; however, ketamine leads to NMDA hyperfunction in PVIs in deep layers. Cotreatment with NAC restores normal NMDA function in both layers. ***A***, Representative traces of NMDA currents (gray) and AMPA currents (black) from recordings in the four treatment groups. NMDAR/AMPAR ratios were recorded in layer 2/3 PVIs held at +40 mV in the presence of picrotoxin; AMPA currents were isolated by bath application of CPP. ***B***, Ketamine treatment resulted in a significant decrease in the NMDAR/AMPAR ratio in layer 2/3 PVIs (*p ≤ 0.05, Bonferroni corrected). This effect of treatment was mitigated by simultaneous treatment with NAC. ***C***, Example traces of NMDA (gray) and AMPA (black) currents from PVIs located in layer 5 of the mPFC. ***D***, Ketamine treatment resulted in a layer-specific increase in the NMDAR/AMPAR current ratios in layer 5 mPFC PVIs. Simultaneous NAC treatment led to a significant decrease in the NMDAR/AMPAR ratio versus animals treated with only ketamine (*p* = 0.035, Student’s *t* test).

The NMDAR hypofunction model of schizophrenia posits that a preferential loss of NMDARs in GABAergic interneurons is a crucial factor in the etiology of the disease (Olney et al., 1999; Nakazawa et al., 2012). However, several recent reports (Jones et al., 2014; Jeevakumar and Kroener, 2016) have provided evidence that perinatal NMDAR blockade actually leads to GluN2B-specific upregulation of NMDARs in PVIs. Because there is also evidence that NMDAR blockade differentially affects the GABAergic networks of the superficial and deep layers of the PFC (Zhang et al., 2008; Jeevakumar and Kroener, 2016), we next compared changes in NMDAR function in PVIs in layers 2/3 and 5, respectively. We first recorded NMDAR and AMPAR currents from layer 2/3 PVIs and compared the ratio of NMDAR to AMPAR currents across our four treatment groups. A one-way ANOVA revealed a main effect of treatment on NMDAR/AMPAR ratios in layer 2/3 PVIs (*F*_(3,22)_ = 3.771, *p* = 0.025). *Post hoc* analysis showed that ketamine treatment significantly lowered the NMDAR/AMPAR ratio in layer 2/3 PVIs compared with saline-treated controls and that NAC treatment prevented this shift (Fig. 6). Thus, contrary to what others have previously observed in layer 5 PVIs (Jeevakumar and Kroener, 2016), developmental ketamine treatment appears to reduce the NMDAR contribution at glutamatergic synapses in layer 2/3 PVIs. To see whether NAC can also affect the ketamine-induced upregulation of NMDAR function in layer 5 PVIs, we next examined NMDAR/AMPAR ratios in layer 5 PVIs. Consistent with the findings by Jeevakumar and Kroener (2016), we also found that PVIs in layer 5 from ketamine-treated animals displayed significantly larger NMDAR/AMPAR ratios and that this trend was reversed in NAC-treated animals (*t*_(10)_ = 2.429, *p* = 0.035; Fig. 6). Together, these findings suggest that NAC is able to prevent synaptic alterations at glutamatergic synapses onto PVIs, regardless of the direction of the shift.

### NAC supplementation during late adolescence reduces mitochondrial ROS and recovers normal inhibition in layer 2/3 pyramidal neurons

**Figure 7.**
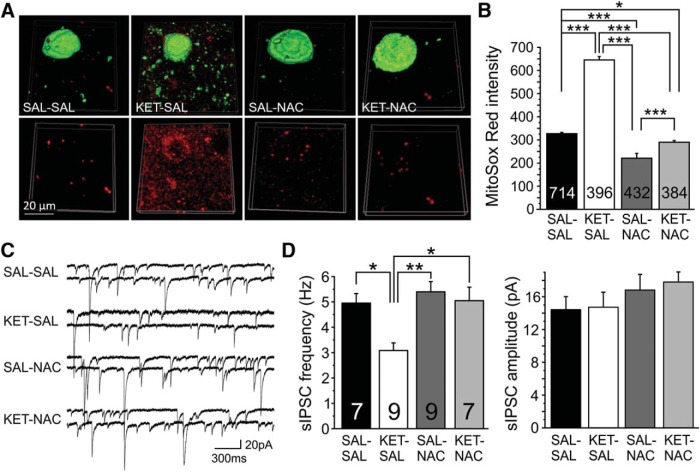
NAC given during late adolescence lowered mitochondrial ROS in PVIs and restored normal inhibition at layer 2/3 pyramidal neurons in mice given perinatal ketamine treatment. ***A***, Following 3 weeks of NAC treatment, acute mPFC slices were collected, stained for Mitosox Red (bottom row), and immunohistochemically colocalized with PV^+^ interneurons (top row, green). ***B***, Animals treated only with KET had a significant increase in mitochondrial ROS within PVIs compared with controls. In contrast, mitochondrial ROS levels in both KET-treated and control animals given 3 weeks of late-adolescent NAC treatment were reduced to levels significantly lower than those in controls. ***C***, Representative traces of spontaneous IPSCs recorded from layer 2/3 pyramidal neurons following late adolescent NAC treatment. ***D***, Animals treated only with KET had a significant decrease in the frequency of sIPSCs onto layer 2/3 pyramidal neurons. Three weeks of NAC treatment was sufficient to recover the frequency of sIPSCs to levels comparable to those in controls. There was no difference in the amplitude of sIPSCs across all four groups. Significance is indicated as *p ≤ 0.05, **p ≤ 0.01, and ***p ≤ 0.001, following Bonferroni correction.

The previous experiments showed that concomitant treatment with NAC during development is sufficient to prevent increases in mitochondrial ROS and disinhibition of mPFC pyramidal neurons in ketamine-treated mice. to determine whether NAC can also protect or restore PVI function when it is given after the critical window of PVI development, we followed perinatal ketamine administration with NAC treatment in late adolescence. In these experiments, NAC was administered for only 3 weeks, starting at P35. In the first experiment, we performed MitoSox Red staining in acute slices and combined this with immunohistochemistry for PV^+^ neurons. A one-way ANOVA revealed a significant effect of treatment on Mitosox Red intensity between groups (*F*_(3,1922)_ = 435.15, *p* < 0.001). Consistent with our previous data, *post hoc* analysis indicated a significant increase in Mitosox Red intensity in animals that received ketamine treatment followed by saline treatment (Fig. 7*A*,*B*). NAC supplementation in late adolescence reversed this effect in ketamine-treated animals and even reduced Mitosox Red intensity below the levels of saline-treated controls. Similarly, control animals given NAC during late adolescence showed a further reduction in Mitosox Red intensity compared with saline-treated controls, suggesting that NAC protected against increases in mitochondrial ROS in PVIs, both under control conditions and during ketamine-induced oxidative stress. Next, we examined whether recovery of mitochondrial function via NAC treatment in late adolescence also restores normal inhibition in the PFC network (Fig. 7*C*,*D*). We recorded spontaneous IPSCs from layer 2/3 pyramidal neurons and compared the frequency and amplitude of events across our treatment groups. A one-way ANOVA revealed a significant effect of treatment on the frequency of sIPSCs (*F*_(3,27)_ = 6.613, *p* = 0.002). *Post hoc* analysis revealed that ketamine-treated animals had a significant reduction in the frequency of sIPSCs compared with controls, which was not present in animals that received NAC in late adolescence. There was no effect of treatment on the amplitude of events (*F*_(3,27)_ = 0.847, *p* = 0.480; Fig. 7*D*). These results suggest that NAC given during late adolescence is able to control rising ROS levels in GABAergic interneurons and to restore inhibition in cortical layer 2/3 pyramidal neurons.

## Discussion

The functional loss of NMDARs in PVIs is thought to underlie the cognitive deficits seen in schizophrenia (Lewis et al., 2005). In rodent models of the disease, perinatal blockade of NMDARs by ketamine causes persistent deficits in PFC-dependent behaviors in adult animals (Jeevakumar et al., 2015). Cognitive deficits include impaired set-shifting ability, abnormal patterns of spontaneous alternation, and deficits in novel object recognition. Developmental ketamine treatment also facilitates behavioral stereotypy in response to an acute amphetamine challenge, which is often used to model positive symptoms of schizophrenia that are associated with a hyperactivated dopamine system (Geyer and Markou, 1995; van den Buuse, 2010), and impairs sensorimotor gating, which is a measure of “pre-attentional” sensory processing (Swerdlow et al., 2001). All of these behavioral abnormalities can be prevented by NAC supplementation during development (Phensy et al., 2017).

Our data add to recent reports showing that developmental rodent models of schizophrenia induce oxidative stress in cortical neurons, leading to reduced GABAergic function in the PFC (Radonji et al., 2010; Johnson et al., 2013; Cabungcal et al., 2014; Jeevakumar and Kroener, 2016), which can be prevented by treatment with NAC (Cabungcal et al., 2014; Do et al., 2015). Ketamine exposure during the second postnatal week resulted in long-lasting changes in oxidative defense systems, which correlated with reductions in PV expression and decreased mitochondrial membrane potential in PVIs of the PFC. Ketamine also increased mitochondrial ROS production in both PVI and pyramidal cells; however, the magnitude of these effects was larger in PVIs. These changes were associated with a decrease in GABAergic inhibition in pyramidal cells and increased glutamatergic inputs in PVIs, which is consistent with an excitatory shift in the E/I balance. In contrast to the upregulation of NMDAR function that occurs in layer 5 PVIs (Jeevakumar and Kroener, 2016), perinatal ketamine treatment caused NMDAR hypofunction in PVIs from layer 2/3, indicating that NMDAR blockade during development induces cell type- and layer-specific changes. All of these deficits were prevented by concomitant antioxidant treatment with NAC. These data highlight the role of mitochondrial dysfunctions in the development of schizophrenia-like symptoms, and they support the idea that NAC administration may counteract the oxidative stress that results from these changes.

Downregulation of GABA release due to NMDAR dysfunction results in the disinhibition of pyramidal cells (Zhang et al., 2008; Kjaerby et al., 2014; Jeevakumar and Kroener, 2016), potentially leading to excessive glutamate release and further oxidative stress (Olney et al., 1999; Behrens et al., 2007). Thus, the reductions in GAD67 and PV levels seen in postmortem brains of schizophrenic patients (Lewis et al., 2005) are believed to represent maladaptive homeostatic changes aimed at maintaining proper E/I balance in the cortical network. While changes in the E/I balance have been extensively linked to critical period plasticity (Hensch, 2005), the mechanisms that control these changes in the developing schizophrenic brain are still not well understood.

Mitochondria are sensors and regulators of intracellular ROS and calcium. Excess cytosolic ROS and calcium elevations compromise mitochondrial reserve capacity, thus promoting mitochondrial ROS generation and release and lowering mitochondrial calcium handling (Brookes et al., 2004). Abnormal mitochondrial function and increased ROS levels lead to faulty ATP production and increase the risk of oxidative damage to lipids, proteins, and DNA that are observed in schizophrenia (Möller et al., 2015). NMDAR antagonists trigger a rapid increase in ROS *in vitro* (Xia et al., 2002; Liu et al., 2013) and *in vivo* (Zuo et al., 2007; Powell et al., 2012). Here we show that subchronic perinatal application of ketamine also leads to long-lasting changes in the glutathione antioxidant defense system and impaired mitochondrial function of both PVIs and pyramidal cells in the PFCs of adult animals. Known effects of ketamine are related to an increase in the proinflammatory cytokine interleukin 6 and activation of the superoxide-producing enzyme NADPH-oxidase (NOX; Behrens et al., 2007; Powell et al., 2012). NOX is a major source of ROS and affects glutamate release in the PFC (Sorce et al., 2010). NOX-dependent oxidative mechanisms have previously been implicated in the loss of PVIs and schizophrenia-like behaviors in different animal models (Schiavone et al., 2009; Cabungcal et al., 2014). NOX and mitochondrial ROS show cross talk that causes mitochondrial dysfunction, leading to a vicious cycle that reinforces intracellular redox imbalance and calcium perturbations (Wenzel et al., 2008; Kröller-Schön et al., 2014). Regulatory redox sites have been found in many proteins involved in glutamatergic neurotransmission. These include the NMDAR itself, in which the oxidation status of a specific redox site on GluN2A subunits regulates the physiologic activity of the receptor (Lipton et al., 2002). Previous reports have suggested that perinatal NMDAR blockade leads to increased NMDAR currents via GluN2B receptors both in adult PVIs in layer 5 of the PFC (Jeevakumar and Kroener, 2016) and layer 4 of the somatosensory cortex (Jones et al., 2014). Our current results show that these effects are layer specific, since PVI in layer 2/3 showed reduced NMDAR currents, consistent with the predictions of the NMDA hypofunction theory of schizophrenia and the observed shift in E/I balance. These distinct changes in glutamatergic signaling might reflect layer-specific differences in the maturation of PVIs and their NMDARs. During development, a shift in NMDAR subunit composition occurs so that the initially high number of synaptic GluN2B subunits decreases, leaving GluN2A subunits, which regulate the expression of PV (Kinney et al., 2006), to predominate in adult animals (Liu et al., 2004; Zhang and Sun, 2011; Paoletti et al., 2013). In pyramidal cells, this switch is at least partly dependent on the activity of the NMDARs (Matta et al., 2011). NMDAR blockade during the second postnatal week likely disrupts this process. Because of differences in layer maturation, the switch from GluN2B to GluN2A signaling likely occurs at different times in layer 2/3 versus layer 5 PVIs. Different ratios of GluN2B and GluN2A subunits may confer different responses to ketamine application, leading to the emergence of NMDAR hypofunction or hyperfunction in PVIs, respectively. The idea that developmental NMDAR blockade induces layer-specific alterations is further supported by the observation that changes in GABAergic transmission seem to be restricted to superficial layers (Kjaerby et al., 2014; Jeevakumar and Kroener, 2016). Lamina-specific alterations in markers of GABAergic interneurons are a prominent feature in schizophrenia (Lewis et al., 2001; Beneyto and Meador-Woodruff, 2008; Beneyto et al., 2011).

Our data also show that early intervention with NAC can prevent the development of mitochondrial dysfunction and synaptic deficits and that even later treatment with NAC may be effective in ameliorating some of the ketamine-induced deficits. *N*-acetyl cysteine has received renewed interest as a potential treatment in a variety of neuropsychiatric disorders (including Alzheimer’s and Parkinson’s diseases, addiction, autism, depression, and schizophrenia) because it interacts with a wide range of factors that contribute to the pathophysiology of these disorders. The targets of *N*-acetyl cysteine include glutamatergic transmission, the glutathione antioxidant system, neurotrophins, apoptosis, mitochondrial function, and inflammatory pathways (Berk et al., 2013; Möller, et al., 2015). Here we focused on the potential antioxidative properties of NAC. *N*-acetyl cysteine is a derivative of the amino acid l-cysteine, which is rapidly oxidized to cystine in the pro-oxidant milieu of the brain; once inside the cell, cystine is reduced to cysteine, which is the rate-limiting substrate for the synthesis of glutathione. Supplementation of NAC is therefore thought to promote the resynthesis of glutathione to neutralize free radicals. Because of its antioxidative and anti-inflammatory properties, NAC has been used as an add-on treatment in schizophrenic patients in whom it improved negative symptoms and global functioning (Berk et al., 2008; Farokhnia et al., 2013). Our data show that concomitant NAC treatment reliably prevents all ketamine-induced changes in the GSH/GSSG ratio and mitochondrial ROS production, as well as the resultant alterations in synaptic transmission. Together, these results suggest an important role of mitochondrial dysfunction in the development of schizophrenia, and they support a concept of early intervention, where prophylactic antioxidant treatment may serve to prevent the development of NMDAR dysfunction and functional loss of PVIs in “at-risk” subjects. However, additional preclinical studies, which may involve pharmacological manipulation of NMDARs during NAC treatment, are required to ascertain that under these conditions NAC affects glutamatergic synaptic transmission primarily through its effects on NMDARs and not through modulation of the cystine–glutamate antiporter, which is another major target of NAC (Berk et al., 2013).

Finally, our data also highlight an important caveat for the clinical use of antioxidants or precursors like NAC: antioxidants can perform differently depending on the cellular milieu, acting either as antioxidant or as pro-oxidant, depending on the redox status of the organism (Harvey et al., 2008; Möller et al., 2015). NAC and glutathione can have both antioxidative and pro-oxidative effects (Sagristá et al., 2002), and in clinical studies the administration of NAC to healthy individuals was shown to decrease their GSH/GSSG ratio (Kleinveld et al., 1992). Some of our measures of PVI function also provide indirect evidence for a potential pro-oxidative effect of NAC under conditions of low oxidative stress. Specifically, PVIs from saline and NAC-treated control mice (SAL-NAC) showed increased levels of ROS (Fig. 3) and received increased numbers of sEPSCs (Fig. 5*B*), indicating changes in E/I function that partially parallel those seen in ketamine-treated animals. Similar trends for increased oxidative stress caused by NAC alone were also seen in a recent influential study from Kim Q. Do’s laboratory (Cabungcal et al., 2014). Thus, while our data strongly support the idea of early intervention, they also underline the need for “early detection” via reliable biomarkers that allow identification of presymptomatic oxidative stress during prodromal stages of schizophrenia to apply antioxidant treatment under the optimal conditions.

## Synthesis

Reviewing Editor: Karen Szumlinski, University of California at Santa Barbara Decisions are customarily a result of the Reviewing Editor and the peer reviewers coming together and discussing their recommendations until a consensus is reached. When revisions are invited, a fact-based synthesis statement explaining their decision and outlining what is needed to prepare a revision will be listed below. The following reviewer(s) agreed to reveal their identity: Christopher Lapish, David Lynch

After consultation with the 2 reviewers of the original manuscript, I am happy to inform you that we all agree that the revised version of your manuscript is very much improved and, with 1 minor revision, is acceptable for publication in eNeuro. Reviewer 2 suggested that one way in which your team could rule out an exclusive involvement of NMDA receptors to your findings would be to test the effects of pharmacological manipulating NMDA receptors and I request that a sentence along these lines be included in the Discussion. Thank you for submitting your work to eNeuro.
